# Identifying Behaviours Representative of Agentic Engagement in Pre-clinical Medical Education PBL Groups Based on Literature and Observations

**DOI:** 10.5334/pme.1414

**Published:** 2024-12-05

**Authors:** Alexandra Androni, Joke Fleer, Laura R. Smids, Joke M. van der Wouden, A. Debbie C. Jaarsma, Johanna Schönrock-Adema

**Affiliations:** 1 University of Groningen and University Medical Center Groningen, Wenckebach Institute for Education and Training, Lifelong Learning, Education and Assessment Research Network (LEARN), Groningen, The Netherlands; 2 University of Groningen and University Medical Center Groningen, Department of Health Psychology, Groningen, The Netherlands; 3 University of Groningen, University College Groningen, Groningen, The Netherlands; 4 Hanze University of Applied Sciences, Groningen, The Netherlands; 5 Utrecht University, Faculty of Veterinary Medicine, Utrecht, The Netherlands; 6 University of Groningen and University Medical Center Groningen, Wenckebach Institute for Education and Training, Lifelong Learning, Education and Assessment Research Network (LEARN), Groningen, The Netherlands

## Abstract

**Introduction:**

In this study we aimed to identify behaviours representative of agentic engagement in pre-clinical medical education problem-based learning (PBL) groups. Agentic engagement is defined as the proactive, intentional contributions students make to their flow of instruction. This concept, developed in secondary education, appears relevant for benefitting optimally from PBL in higher medical education.

**Methods:**

We followed a four-step process to identify any behaviours representative of agentic engagement in our PBL setting. Following a literature search on agentic engagement scales, proactive behaviour, PBL practices and adult learning, we listed behaviours that could denote agentic engagement in our context. We fine-tuned this list through exploratory observations and tailored it to our specific context of second-year PBL groups.

**Results:**

We identified ten observable student behaviours representative of agentic engagement within medical PBL groups. Some aligned with previous literature (asking questions, telling the teacher what they (dis)like, telling the teacher what they are interested in, defending opinions, expressing expectations, suggesting co-operation), and others had not been identified before as representative of agentic engagement (making learning as interactive as possible, creating alternative ways of covering the material, correcting content and enriching others’ insights); thereby introducing novel behaviours unique to our setting.

**Discussion:**

In medical PBL groups, we identified both known agentic engagement behaviours and distinctive behaviours specific to our context, thereby highlighting that the expression of agentic engagement is context-bound.

## Introduction

Medical education programmes and, hence, medical students have to deal with an ever growing amount of clinical knowledge and insights [1]. Additionally, medical students often face high academic demands [2]. Therefore, it is important for medical students to manage the study matter they need to master and the learning outcomes they strive to achieve in ways that best work for them [3]. Personalizing their learning through agentic engagement, a form of student engagement that has recently been introduced in the literature, may help mitigate the pressures resulting from their studies and aid in sustaining medical students’ drive: personalizing their education may resemble tailor-made teaching and as such, may make learning more relevant and more motivating.

Student engagement is a useful perspective for medical educators seeking to understand how and why medical students react and learn in diverse learning activities. In academic work, student engagement is defined as students’ psychological investment in learning and mastering the skills that the learning process promotes [4]. Until 2011, the student engagement construct was conceptualized as being three-dimensional, containing a behavioural (implying students’ effort and attention), a cognitive (students’ sophisticated rather than superficial learning strategies) and an emotional (presence of interest and enthusiasm) dimension [5]. This tri-fold conceptualization described students’ reactive engagement to teacher-provided material. Yet it failed to represent the extent to which students contribute to, modify and enrich their learning processes [6]. Such disparity between reactive and proactive learning prompted the addition of agentic engagement as a fourth component of student engagement in 2011 [7].

Agentic engagement refers to how students actively contribute to the flow of instruction they receive [7]. It produces two important outcomes: optimized learning conditions and improved student functioning [8]. Students who are agentically engaged not only perform academically, but ‘create, enhance, and personalise the conditions under which they learn’ [7]. In line with the aforementioned engagement components, agentic engagement allows students to direct their own learning but differs at being influencing rather than reactive to the learning activity [9] and more easily observable. Further research [6] pinpoints the subtle difference of how agentic engagement is described as something that happens in the presence of a teacher but does not reside within the student; instead, it is externalized. That is to say, students speak up about their instruction to improve the learning process instead of accepting conditions as they are (i.e., when the teacher provides information for students to comprehend, agentically engaged students will verbally communicate ways to enhance the learning experience). Drawing upon Reeve and Tseng [7], Wakefield [6] outlined five characteristics as key to agentic engagement: in order to qualify as agentic engagement, behaviours must be constructive, contributional, proactive, intentional and influencing. Aside from this, the most frequently observed agentic engagement acts include: students communicating their preferences, expressing their feedback and offering suggestions with respect to the learning process [9,10].

Until recently, agentic engagement has mainly been studied in a secondary school context, where the flow of instruction is predominantly created by the teacher [9]). In secondary schools, pupils often need explicit and detailed instruction because they are still developing their self-regulation skills [11]. This teacher-centred approach can allow limited room for student engagement [12] and task exploration [13] because of (task-)specific learning directives (i.e., how to study effectively and when to use which learning strategies). In the higher medical educational context, students have shown advances in self-control, risk-taking and willingness to seek new challenges, which implies that they are better prepared to navigate the increased autonomy in their learning processes, promoting the cultivation of a lifelong learner mindset [14]. As a student-centred educational approach may foster these developments, teachers take on a co-regulating role, guiding the instruction through questioning rather than providing direct instruction [15]. By explicitly paying attention to agentic engagement, higher education institutes may support the cultivation of lifelong learning even more by offering students additional room for choice and leadership.

To support students as independent adult learners, problem-based learning (PBL) has been implemented in higher medical education as a student-centred approach. PBL is a small-group based approach that relies on independent study and collaborative analysis of investigating problems [16], rather than being directly instructed by teachers. Students in PBL apply “cognitive, affective and behavioural components providing them with the capacity to achieve desired results in light of changing environmental conditions” [17]. As such, PBL embodies fundamental elements of agentic engagement in terms of: a) students taking responsibility for their learning, b) acquiring skills through inquiry, and c) choosing learning resources and implementing appropriate strategies rather than relying on teacher-supplied knowledge. In order for educators to facilitate an environment that allows students to engage agentically, it is important to understand how students in higher medical PBL education display their agentic engagement; how they try to optimize their learning conditions. Such acts may be expressed by agentic engagement strategies different from those in teacher-centred, secondary education, considering the needs and abilities of students in either educational tier [18,19]. While PBL inherently nurtures agentic engagement principles, agentic engagement adds value to and strengthens the core qualities of PBL by *personalizing* one’s learning [20], which is crucial in the highly dynamic field of higher medical education.

Building on these insights, the aim of this study was to identify behaviours representative of agentic engagement in a higher medical education PBL setting. Considering that higher education students are more adept at handling autonomy in their learning processes and have increased opportunities for agentic engagement, we anticipated a wider range of agentic engagement behaviours compared to those observed in secondary education. Consequently, we sought to explore how agentic engagement manifests in our context. The question this study aimed to answer was: what behaviours are indicative of agentic engagement in a PBL higher medical education setting?

## Methods

We employed pragmatism as our epistemological approach, based upon its premise of utilizing the best methods to investigate real-world problems, allowing for the use of multiple sources of data and knowledge to answer research questions [21]. Pragmatism does not necessarily require one particular method or combination of methods. Rather, it aims to simply address the research question, investigate a phenomenon, or test a theory with the most appropriate research method(s) [22].

### Educational context

This study was performed in undergraduate medical education at the University Medical Center Groningen (UMCG). Part of the undergraduate PBL curriculum includes tutor groups, which constitute compulsory contact hours and focus on patient-centred complaints. Students attend two tutor sessions a week with a duration of two hours, and the groups consist of seven to twelve students and one tutor. A tutor is present in order to give feedback and explain tasks.

The tutor’s role can best be characterized as that of a ‘process supervisor’ who supervises the learning process. Moreover, the tutor serves as a ‘role model’, in the sense that they have progressed further in the course than the students and are already working as a young medical professional – a position that the students will find themselves in in a few years’ time. The tutor’s duties are limited to ensuring that students cover the week’s material by forming relevant questions and they interrupt the group session only where necessary. Simultaneously, the tutor monitors that the students elaborate on the material in sufficient depth. During the first session, students are presented with a patient case and discuss possible diagnoses and/or therapeutic strategies on the basis of the curriculum’s learning outcomes. The summary of the patient case during the tutor group session is done by a student appointed as the treating physician (TP) of the week. The TP is also the ‘person in charge’ of resolving problems of the patient that arise throughout the case. The homework consists of finding answers to the questions formulated during the first tutor group session, which are then shared during the following group meeting through oral presentations.

### Participants

We invited seven tutor groups enrolled in second-year pre-clinical medical education. We deliberately chose second-year students, as they, unlike first-year students, are already familiar with the structure of PBL and the purpose of tutor group sessions, and because third-year sessions take place without tutors, making them less suitable for studying agentic engagement.

Tutors were emailed and invited to participate in the study after reading the information letter. If a tutor did not consent to participate, a tutor from another group was invited. If the tutor and all students from their tutor group gave permission to participate, we asked them to sign the informed consent form mentioning their participation and their permission for the sessions to be videotaped. If at least one student did not agree to be part of the study, a new group was invited to participate. We conducted our study in agreement with the declaration of Helsinki with approval by the Ethics Review Board of the Netherlands Association for Medical Education (NVMO), dossier number: 2019.2.11.

### Identification of agentic engagement

We studied agentic engagement in a stepwise process consisting of four steps, namely 1) a literature search; 2) listing potentially relevant literature-sourced observable agentic engagement behaviours; 3) exploratory observations; and 4) identification of agentic engagement behaviours. Overall, we explored some specific theories that might lead us to behaviours indicative of agentic engagement. We based the choice of such theories on our research team’s expertise in the field of psychology and education research and used these to snowball search and generate input for our draft observation list. We then complemented the list with behaviours from our observations.

### Step 1: Literature search

Drawing upon Reeve and Tseng’s [7] definition of agentic engagement, we first searched for existing agentic engagement measuring tools [7,23]. Furthermore, on the basis of our setting being one of a student-centred, constantly enquiring nature, we conducted a focused literature review on closely related concepts. These included students’ proactive behaviour [24,25] PBL characteristics [18,26,27,28] and adult learning practices used by students in medical education [29,30] that could serve to identify agentic engagement behaviours relevant to our context. Such student behaviours needed to show analogies with the conceptualization of agentic engagement as to how students use their freedom of action to personalize their learning and make it more meaningful.

### Step 2: Listing potentially relevant literature-sourced observable agentic engagement behaviours

To ensure that our literature-sourced behaviours could be practically identified through observation in the next steps, we used Wakefield’s [6] five characteristics of agentic engagement as criteria to determine whether an externalized observable behaviour represented agentic engagement. Agentic engagement needed to involve verbal cues and reflect constructive, contributional, proactive, intentional and influencing behaviours. We then drafted a list of all agentic engagement behaviours that we identified as potentially relevant to our context. When necessary, we changed the wording of behaviours to tailor it to our study context by reformulating the statements sourced from literature.

### Step 3: Exploratory observations

To assess the practical feasibility of our list of potential agentic engagement behaviours, we applied it to medical tutor groups at the UMCG. Two members of the research team (LRS, AA) used the list as a template to analyze student behaviours during two tutor sessions of each group. They video-taped and audio-recorded all sessions using two cameras from two different views to include all students on tape. Before starting the recordings, they randomly assigned a capital letter tag to all students and tutors in the observed tutor group. By noting the letters corresponding to the student/tutor behaviour observed, these behaviours were easily identifiable when re-visiting the recorded observations. Both researchers attended the first four sessions, noting down students’ observable agentic behaviours. This way, we were able to get a good understanding of the classroom environment, non-verbal reactions and to achieve agreement between the two of us when observing student behaviour. One researcher attended and videotaped five out of the remaining ten sessions, while the other researcher handled the remaining five sessions. The researcher who attended the sessions initially analyzed them directly, and the other researcher, who did not attend the sessions, analyzed them by watching the corresponding video recordings. After both researchers (LRS, AA) analyzed all sessions, they merged their copies of the draft list. The researchers presented behaviours recognized as agentic engagement by both, along with a number of cases where there was uncertainty about whether they constituted agentic engagement, to the research team to ascertain which contributions represented agentic engagement and which did not.

### Step 4: Identification of agentic engagement

Behaviours included in the draft list were not always indicative of agentic engagement. For example, a behaviour like *“student asks questions”* was not always intended to influence the flow of instruction (Table 1). After reaching consensus on *when* certain behaviours embodied agentic engagement and *when not*, we were able to determine which observed behaviours were representative of agentic engagement, which not and which were not observed during our observations.

**Table 1:** Examples of when certain items were considered agentic engagement and when not, based on whether the respective student behaviour constructively influenced the flow of instruction.


ITEM DENOTING AGENTIC ENGAGEMENT	EXAMPLE OF AGENTIC ENGAGEMENT	EXAMPLE OF NO AGENTIC ENGAGEMENT

Student tells the teacher what they (dis)like	e.g., “I really enjoy watching videos of how the gluteus goes into flexion, especially in slow-motion videos with athletes. Please use more of these next week”	e.g., “I really don’t like it when they give us 80 pages to study in one week and we also have to prepare for the consultation skill test”

Student asks questions	e.g., “I read that after colon surgeries, patients can still have an intact anal canal. What does this mean?”	e.g., “What time does the anatomy practical take place next week?”


## Results

### Step 1: Literature search

Our initial literature search yielded two validated agentic engagement scales (Agentic Engagement Scale (AES) [7], Enlarged AES [23]), and individual student practices describing proactive behaviour, PBL characteristics and adult learning in medical education. Specifically, this search resulted in 18 individual student practices potentially reflecting agentic engagement (see step 1, Table 2).

**Table 2:** Overview of student behaviour studied in the identification of agentic engagement.


STEP 1: RESULTS OF LITERATURE SEARCH PRACTICES		STEP 2: LISTING POTENTIALLY RELEVANT LITERATURE-SOURCED OBSERVABLE AGENTIC ENGAGEMENT BEHAVIOURS (AFTER ADJUSTMENT OF THE WORDING)	STEP 3: EXPLORATORY OBSERVATIONS		STEP 4: IDENTIFICATION OF AGENTIC ENGAGEMENT	

			BEHAVIOUR	REPRESENTATIVE QUOTE		

Agentic Engagement Scale (AES)	(1) During class, I ask questions^1^ (2) I tell my teacher what I like and what I don’t like^1^ (3) I let my teacher know what I’m interested in^1^ (4) During class, I express my preferences and opinions^1^ (5) I offer suggestions about how to make the class better^1^	(1) Student asks questions (2) Student tells the teacher what they (dis)like (3) Student tells the teacher what they are interested in (4) Student expresses preferences and opinions (5) Student offers suggestions about how to make the class better	(1) Student asks clarifying questions adding to the flow of instruction (2) Student tells the teacher what they (dis)like • *Behaviour 2(3) not observed*- • *Behaviour 2(4) removed*- • *Behaviour 2(5) removed*-	(1) *“Could you please explain which is the dorsal side of skull, and what exactly is the difference between posterior and dorsal?”* (2) *“I like practicing with previous exam questions as preparation. Could you discuss some with us the week before the exam?”*	(1) Student asks clarifying questions adding to the flow of instruction (2) Student tells the teacher what they (dis)like (3) Student tells the teacher what they are interested in	Characteristics: Student-Initiated/Teacher-Prompted and Unilateral/Transactional

Enlarged AES	(6): During classes, I introduce new issues or discussion topics (student contributes unilaterally)^2^ (7) I defend my opinions even if they are not in line with those of my classmates^2^ (8) Student contributes transactionally by questioning and involving the teacher^2^	(6) Student contributes unilaterally by introducing new discussion topics (7) Student defends their opinions (even if not in line with peers’) (8) Student contributes transactionally by questioning and involving the teacher	• *Step 2; Behaviour 2(6) removed*- (3) Student enriches others’ insights (new behaviour) • *Behaviour 2(7) not observed*- • *Behaviour 2(8) removed*-	(3) *“You could always remember diaphragm paralysis from the mnemonic C3 to C5 keeps the diaphragm alive”*	(4) Student enriches others’ insights (5) Student defends their opinions (even if not in line with peers’)	

Literature on agency and proactive behaviour	(9) Student expresses expectations^3^ (10) Student takes uninvited initiative or steps in for another^4^	(9) Student expresses expectations in line with the learning material (10) Student takes uninvited initiative or steps in for another	(4) Student expresses expectations in line with the learning material (5) Student corrects the given content (new behaviour)	(4) *“It’s important to know common joint injuries and because there are about 7 mentioned in Kumar, maybe we should have 2 people present those”* (5) *“No, it doesn’t work like that. Your employer pays for a maximum of 2 years and then you may be eligible for a WGA benefit”*	(6) Student expresses expectations in line with the learning material (7) Student corrects the given content	

Literature on PBL theories	(11) Student raises their hand^5^ (12) Student chooses to sit next to the educator^6^ (13) Students seeks information on their smartphone/laptop^7^ (14) Student walks around the classroom^7^ (15) Student seeks information in their book^8^	• *Step 1; Behaviours 1(11) to 1(15) were removed*-				

Adult learning theories in medical education	(16) Student recommends a theme which they want to work on^9^ (17) Student suggests co-operation with another student or proposes teamwork^9^ (18) Student tries to make class more interesting^10^	(11) Student recommends a theme which they want to work on (12) Student suggests co-operation with another student or proposes teamwork (13) Student tries to make learning more interesting	(6) Student creates alternative ways of going through the learning material (7) Student suggests co-operation with another student or proposes teamwork (8) Student makes learning as interactive as possible	(6) *“Since we have a lot of anatomy this week, let’s add a video to the presentation so it doesn’t get too boring”* (7) *“[…] and you 3 can work together to make questions on the national vaccination policy”* (8) *“Varum: imagine riding a bicycle (your legs spread, and Var-room-room!). Valgum: chewing gum, knees attached to each other”*	(8) Student creates alternative ways of going through the learning material (9) Student suggests co-operation with another student or proposes teamwork (10) Student makes learning as interactive as possible	


^1^ AES, Reeve & Tseng (2011), ^2^Mameli & Passini (2018), ^3^Cohen (2020), ^4^Murdoch-Eaton & Whittle (2012), ^5^Roy (2017), ^6^Reimschisel (2017), ^7^Cho (2017), ^8^Reed et al. (2014), ^9^Taylor & Hamdy (2013), ^10^Brydges & Butler (2011).

### Step 2: Listing potentially relevant literature-sourced observable agentic engagement behaviours

We then cross-checked whether the 18 literature-sourced behaviours from step 1 met Wakefield’s [6] criteria of being constructive, contributional, proactive, intentional and influencing. We purposefully removed behaviours related to gestures, sitting positions and techniques of seeking information (see Table 2; Step 1; 11–15) because the underlying motives or reasons for these behaviours were not visible or verbalized, implying that these behaviours did not clearly adhere to Wakefield’s criteria. As a result, our literature-based draft list included 13 behaviours that we judged potentially relevant for identifying agentic engagement in our medical PBL setting. Of these, we reformulated student behaviours listed in the literature as self-reported practices into observable behaviours. For example, we adjusted the original act “*I tell the teacher what I like*” into “*Student tells the teacher what they like*”. The resulting draft list with 13 behaviours can be found in Table 2, Step 2.

### Step 3: Exploratory observations

From the seven tutor groups that we initially invited to participate in the study, two groups refused to participate. In one of these groups, the tutor declined our invitation, and in the other, a student did not consent. The two tutor groups that we additionally approached agreed to take part in the observation study, so that, in total, seven tutor groups participated in our study. The (recorded) observations comprised fourteen meeting video-recordings, totalling sixteen hours, thirty-four minutes, and twenty-eight seconds (16:34:28) of video-recorded data.

Based on the 13 behaviours listed as potentially relevant to denote agentic engagement, observations, we observed three behaviours in the form that were initially proposed in previous literature. These were when a student 1) tells the teacher what they (dis)like, 2) expresses expectations in line with the learning material, and 3) suggests co-operation with another student or proposes teamwork.

Next, we adapted three behaviours of the preliminary list based on our observations. Firstly, we reformulated “Student asks questions” (Table 2; Step 2; behaviour 1) into “Student asks clarifying questions adding to the flow of instruction” since it was only the latter that resulted in a constructive change. Secondly, we modified “Student recommends a theme which they want to work on” (Table 2; Step 2; behaviour 11) into “Student creates alternative ways of going through the learning material” because students in our medical PBL groups both recommended a learning theme to cover *and* suggested alternative ways of studying the learning themes, but it was only the latter behaviour that represented agentic engagement during the tutor group meetings. Thirdly, we rephrased “Student tries to make learning more interesting” (Table 2; Step 2; behaviour 13) to “Student makes learning as interactive as possible”, seeing how all propositions to make class more interesting involved interactive quizzes, polls and whole-class games.

Additionally, there were two behaviours we did not encounter in classroom observations. These were “Student tells the teacher what they are interested in” (Table 2; Step 2; behaviour 3) and “Student defends their opinions (even if not in line with peers)” (Table 2; Step 2; behaviour 7).

We removed four behaviours from the list based on the observational material. We removed “Student expresses preferences and opinions” (Table 2; Step 2; behaviour 4), because we believed this was covered by “Student tells the teacher what they (dis)like” (Table 2; Step 3; behaviour 2) and by “Student expresses expectations in line with the learning material” (Table 2; Step 3; behaviour 4). We also eliminated “Student offers suggestions about how to make the class better” (Table 2; Step 2; behaviour 5) because we agreed that this was too abstract and was covered well by other behaviours on our list. Two more behaviours we removed were unilateral and transactional contributions (Table 2; Step 2; behaviours 6 and 8). The reason for this was that these were more applicable to the processes than the behaviours themselves. We made this decision after observing how students contributed unilaterally not only when introducing new discussion topics (Table 2; Step 2; behaviour 6), but also when being agentically engaged otherwise, for example when asking questions or expressing expectations. Likewise, students contributed transactionally not only when questioning and involving the teacher (Table 2; Step 2; behaviour 8) but also by questioning and involving their peers. In other words, these terms did not describe only one agentic behaviour, but multiple.

Finally, we recognized two novel agentic engagement behaviours and added these to our list. One was “Student enriches others’ insights” (Table 2; Step 3; behaviour 3). We observed students in tutor groups sharing valuable ideas or clinically-orientated knowledge that had greatly benefitted their understanding of the study material. These behaviours always denoted agentic engagement by supplementing the instruction.

*Example (Student enriches others’ insights)*

A student took initiative by sharing her own mnemonic that helped her remember the leg motions mentioned by the student presenter. After sharing this, another student asked her to repeat her mnemonic. All students took notes of her mnemonic, while one of the students asked if this could also be applied to the motion of the toes.

A second novel agentic engagement behaviour was “Student corrects the given content” (Table 2; Step 3; behaviour 5). Although this resembled when a “Student takes uninvited initiative or steps in for another” (Table 2; Step 2; behaviour 10), students were observed interrupting the learning and constructively intervening, by correcting the (incorrect) content that was previously shared (by their peers).

*Example (Correcting the given content)*

In a discussion on social healthcare, the tutor asked if the students are aware of the Ministry of Health welfare benefits. One student answered: “The IVA-benefit *(Disabled Workers Income Scheme)* and the WGA-benefit *(Return to Work Scheme for the Partially Disabled)* are for people who can’t work for up to 2 years after their disability”. Then, another student who was more certain of the distinction, joined the conversation and exclaimed “No, this is not how it works. Your employer will pay for maximum 2 years and then you may be eligible for a WGA-benefit.”

To conclude, we observed eight agentic engagement behaviours in our medical PBL classes. These can be viewed in Step 3 (Table 2).

### Step 4: Identification of agentic engagement

Out of the initial 18 behaviours on our draft list of potential agentic engagement indicators, we retained 10 agentic behaviours that were representative of agentic engagement in medical education PBL classes (Table 2, Step 4).

#### Closer inspection of observational data

Our observations on medical student agentic engagement showed how agentic engagement was not always student-initiated [7]. We observed how students engaged agentically not only by taking their own initiative, but also in response to their tutor’s encouragement to participate constructively in the learning and to thereby influence it. We differentiated agentic behaviours between student-initiated and teacher-prompted.

- *Student-initiated*: Agentic engagement behaviour without the tutor’s invitation to participate (students engage by own initiative).
- *Teacher-prompted*: Agentic engagement behaviour displayed after a tutor’s invitation to participate in class.

To illustrate, we added these two terms to the identification of agentic engagement in PBL small group classes (Table 2, Step 4, characteristics). Similarly, we distinguished between unilateral and transactional agentic engagement.

- *Unilateral:* agentic engagement describing individual students’ contributions to the learning process,
- *Transactional:* agentic engagement describing multiple students’ contributions towards a common dialogue between their peers/tutor.

We hereby present this differentiation between behaviours and characteristics of agentic engagement. This way, the terms unilateral/transactional and student-initiated/teacher-prompted may describe more than one agentic engagement behaviour. A complete overview of the identified agentic engagement behaviours can be seen in Table 2.

## Discussion

The purpose of this study was to identify student behaviour representative of agentic engagement in a higher education medical PBL setting. Specifically, we focused on whether and how agentic engagement could be observed in pre-clinical small group medical classes. We identified 10 individual student behaviours that we considered representative of agentic engagement in higher medical PBL education. Our findings indicate that agentic engagement cannot be generalized universally across all situations or environments. Instead, it is shaped and defined by the particular context in which it takes place.

The expression of agentic engagement in our context differed notably from secondary education contexts for which agentic engagement was originally operationalized [9]. Our work adds to the literature of agentic engagement in several ways. Firstly, our study expands earlier agentic engagement work [9,23,31], by introducing students’ personal and complementary additions such as enriching others’ insights or creating alternative ways of going through the learning material. In other words, we observed students making proactive and intentional contributions to optimize their learning processes by intervening with their own expertise rather than merely contributing to it. Secondly, we propose the term ‘transactional contribution’ to refer to students’ agentic engagement involving more than one student’s agentic participation, instead of using this to describe contributions towards classmates or towards the teacher as suggested by Mameli and Passini [23]. Our observations support the ‘unilateral’ nomenclature, where an individual student directs their agentic contribution towards the rest of the group (peers or tutor) without it being followed by further student engagement. A third novel finding of our study is that of agentic engagement aimed towards peers and not just towards the tutor. A number of the student behaviours (i.e., asking questions, communicating likings, expressing interests) were consistent with previous secondary schooling conceptualizations [9,18,32] of students’ agentic engagement upon teacher’s instruction. However, we often observed how students in the group automatically used their peers (presenting or acting as the TP) as a teacher substitute, given how they were also an ‘expert’ on the subject when presenting to their study group. This implies peer-assisted learning, a critical parameter in medical education leading to improved student learning [33] and enhanced student motivation [34]. Fourthly, we identified agentic engagement not only as a student-initiated [11,35], but also as a teacher-prompted pathway. This suggests that teachers can stimulate agentic engagement from their end and that they could be trained to do so. Asking for students’ perspectives and fostering students’ needs for autonomy during learning activities has been previously described as autonomy-supportive teaching [11,36] and our identified behaviours can serve as a starting point towards providing more, long-lived autonomy-supportive teaching.

To the best of our knowledge, this is the first study that explored the observable expression of agentic engagement in a higher medical education setting. Our work may provide a foundation for educational researchers to identify agentic engagement in PBL higher education contexts in a way that goes beyond the original self-reported scales [9,23] by involving group observations. After all, observational methods provide an advantage when considering the nature of agentic engagement being collaborative and requiring others to facilitate learning [6]. We built on prior research by applying agentic engagement insights to a broader context beyond secondary education. This effort resulted in a comprehensive identification of agentic engagement within the higher medical PBL education environment, informed by literature-derived learning practices characterizing students in higher medical PBL education and by observations in PBL groups.

### Limitations

Despite aforementioned strengths, our study has several limitations. First, although we performed a literature search guided by the expertise of our research group, focusing on relevant agentic engagement publications and related constructs, there is a possibility that we may have overlooked some relevant publications. Secondly, our identification of agentic engagement is limited to observable student behaviours on a whole-classroom, group level. Therefore, we do not have insights into students’ agentic engagement behaviours outside the classroom nor into their individual inclinations to demonstrate agentic engagement. A group-expression of agentic engagement might have masked agentic engagement behaviours on an individual student level. In other words, once a single student engaged agentically within the group, this relieved the need for several other students to do the same. Hence, while using peers as proxies can promote collaborative learning and peer support [37], it can also mitigate individual students’ agentic contributions. Not having observed agentic engagement in certain students does not necessarily imply that they lack a propensity for displaying this engagement [38]. Thirdly, we also grasp that the presence of cameras may have affected students’ behaviours by discouraging them to exhibit initiative they would have otherwise showed, for instance because of insecurity/lack of self-efficacy. In that case, we may have missed out on relevant agentic engagement behaviours. However, recording the tutor group sessions could have also elicited additional agentic engagement behavior, which we grant as a positive consequence in our study. Lastly, the results of our study may not be generalizable because all sessions observed took place in one medical school and with a limited number of students. Moreover, student agentic engagement levels vary from meeting to meeting, posing challenges to extrapolating our specific observations to broader contexts.

As we limited our study to observable behaviours within pre-clinical medical education, further research is needed to explore its applicability to non-PBL medical education contexts (i.e., lecture-based learning) or to higher non-medical PBL education contexts. Despite this, our identifications can help institutions offering PBL medical education understand how students engage agentically in class and how educators can further stimulate this engagement.

#### Recommendations

Based on our study findings, we propose an adaptation of the definition of agentic engagement. This adaptation moves away from the conceptualization of students’ *contributions into the flow of instruction they receive* [7]. To our understanding, a ‘flow of instruction’ suggests teacher-centred scaffolded direction towards students’ learning, which students react to. Such learning conditions may foster agentic engagement by welcoming choices and taking students’ preferences into account [20,39]. However, in our higher medical education PBL context, students engage agentically in more ways than solely contributing to the flow of instruction, partly because teacher-provided instruction is minimal in this setting and often replaced by peer collaboration. In fact, students engage agentically *to personalize and optimize their learning conditions and experiences*. This formulation also captures what agentic engagement entails in the secondary education context. In this manner, students’ agentic engagement in PBL ‘improves their functioning and their learning circumstances’ [8]. Therefore, we recommend the following working definition of agentic engagement in PBL and similar student-centred educational settings: *agentic engagement is students’ proactive involvement, through explicit communication, in personalizing and optimizing their learning conditions and experiences consistent with their needs, preferences, and abilities.”*

#### Implications for practice and future research

We compiled an observation sheet of behaviours that we identified as embodying agentic engagement in the context of pre-clinical medical education. We can envision that, in future research, this agentic engagement list serves as a basis for measuring agentic engagement behaviours in higher education students seeking to optimize their learning processes. Considering our finding that educators can encourage students to exhibit agentic engagement, this agentic engagement list may also serve to guide and enhance educator training, in order to help them better recognize and stimulate agentic behaviours in students [8]. Being aware of agentic engagement expression within their teaching, educators participating in PBL classes can thereupon instantly adjust their learning environment to more effectively stimulate students’ agentic engagement.

Additional research is required to assess the comprehensiveness of our proposed agentic engagement list. It is also necessary to further explore whether agentically engaged students employ other, unobservable practices to optimize their learning conditions, or whether they engage agentically outside the classroom. Future research can also focus on the factors (de)motivating students to engage agentically in group classes and the teacher’s role in this. Are all agentic behaviours effective in improving students’ learning processes? Can tutors promote agentic engagement by interfering less during small group meetings? Further recommendations include the exploration of the above presentations of agentic engagement through personal student interviews or focus group discussions. Observational material may in these instances serve as a partial reference in interviewing students and inquiring on their agentic engagement depending on the content, peers and tutor. This way, not only students who displayed agentic engagement can be asked about their personal goals and ways of showing engagement, but student ‘bystander’ behaviour can also be examined as to when and how these subgroups engage agentically, and what factors contribute to their (dis)engagement. Further research is essential to determine the suitability of our list of agentic behaviours. This research should include an examination of the construct validity of our identification tool when applied in practical settings. Specifically, it is crucial to investigate whether this tool can reliably capture and report students’ agentic engagement in small-group PBL classes.
